# Associations Between Self‐Reported Dietary Intake and Atopic Dermatitis Risk in Young Adults From Singapore and Malaysia

**DOI:** 10.1111/cea.14629

**Published:** 2025-01-21

**Authors:** Jun Jie Lim, Kavita Reginald, Yee‐How Say, Mei Hui Liu, Fook Tim Chew

**Affiliations:** ^1^ Department of Biological Sciences, Faculty of Science National University of Singapore Singapore Singapore; ^2^ Department of Biological Sciences, School of Medicine and Life Sciences Sunway University Petaling Jaya Malaysia; ^3^ Department of Biomedical Science, Faculty of Science Universiti Tunku Abdul Rahman (UTAR) Kampar Malaysia; ^4^ Department of Food Science & Technology, Faculty of Science National University of Singapore Singapore Singapore

**Keywords:** atopic dermatitis, dermatology, dietary intake, education, epidemiology, macronutrients, prevention

**Summary**

- Occasional high‐fat and high‐protein food intake may be associated with a lower odds of AD.
- A balanced, occasional intake of fats, proteins and GI may offer a potential strategy for managing AD.

To the Editor,

Atopic dermatitis (AD) is a chronic skin condition that impacts patients' quality of life, requiring long‐term management strategies. As fundamental components of the human diet, macronutrients can affect inflammatory responses, especially when consumed excessively or imbalance, potentially exacerbating AD. Thus, dietary modification may be a viable complementary management strategy. However, the lack of clear guidance and limited research on optimal macronutrient intake complicates AD management. Using our derived frequency‐based dietary indices [1, 2, 3], we investigated the association between macronutrient intake and current AD in a well‐defined cohort from Singapore and Malaysia. These indices assess overall diet quality based on the intake frequency of high‐fat, high‐protein and high‐glycaemic index foods, offering valuable insights into how dietary habits may impact AD risk.

Additional Information about study methods and findings are available in the following repository (https://osf.io/35q42/?view_only=02a946147bce4ca3b2a524700f5043a2). Our study drew from the Singapore/Malaysia Cross‐sectional Genetics Epidemiology Study (SMCGES) cohort (2005–2023), conducted in adherence to the Declaration of Helsinki and Good Clinical Practices and in compliance with local regulations. Ethical approval was granted by NUS Institutional Review Board (Reference Codes: NUS‐07‐023, NUS‐09‐256, NUS‐10‐445, NUS‐13‐075, NUS‐14‐150, NUS‐18‐036) and ethics committees from Universiti Tunku Abdul Rahman (UTAR) (Ref. U/SERC/03/2016) and Sunway University (Ref. SUREC 2019/029), respectively. Details of SMCGES have been previously reported [1, 2, 3]. All participants gave informed consent. Data were collected using a standardised questionnaire based on the International Study of Asthma and Allergies in Childhood (ISAAC) protocol, covering atopic medical histories, socioeconomic factors, lifestyles, dietary habits and anthropometrics. The cohort consisted mainly of university students, with a mean age of 23.0 years (SD ± 6.34) and a mean body mass index (BMI) of 20.6 kg/m^2^ (SD ± 4.35).

In this cross‐sequential study, atopy was defined as a genetic predisposition to immediate hypersensitivity against environmental allergens, assessed via skin prick test (SPT) responses to house dust mite allergens, *Blomia tropicalis* and *Dermatophagoides pteronyssinus* [4]. A positive SPT (62.4%) indicated personal atopy. Current AD (*n* = 2729, 16.0%) was diagnosed using the UK Working Party and Hanifin‐Rajka criteria, which required both clinical symptoms and atopic sensitisation [5, 6]. Specifically, individuals with recurrent, itchy flexural rashes lasting at least 6 months within the past 12 months, along with a positive SPT response, were classified as having current AD. Clinical assessments were performed by trained personnel and cross‐verified by a dermatologist. Atopic sensitisation is a key criterion for diagnosing AD, considered alongside clinical symptoms to ensure alignment with established guidelines.

Dietary habits were assessed using a validated, semi‐quantitative food‐frequency questionnaire (FFQ), adapted from the ISAAC Phase III protocol, covering intake frequency for 16 food groups [7]. Designed for large‐scale global studies, this FFQ offers a cost‐effective and practical method for capturing essential dietary data in settings with many participants. It has been widely used and cross‐validated within the ISAAC framework, demonstrating its relevance for identifying associations with allergic diseases [7]. The method for calculating dietary indices has been described previously [1, 2, 3].

Logistic regression, adjusted for age, sex, BMI (Asian class), parental AD and education, alcohol use and energy intake, showed that a dose‐dependent association between lower intake of high‐fat, protein and GI foods and lowered odds of current AD among 17,060 participants. Multiple comparisons and Type I errors were controlled using the False Discovery Rate method. Lowering all three dietary factors was significantly associated with reduced AD odds, with infrequent intake of high‐fat (Adjusted odds ratio [AOR]: 0.625; 95% Confidence Intervals [CI]: 0.544–0.718; adjusted *p*‐value: < 0.001), high‐protein (AOR: 0.620; 95% CI: 0.548–0.701; adjusted *p*‐value: < 0.001) and high‐GI foods (AOR: 0.656; 95% CI: 0.575–0.749; adjusted *p*‐value: < 0.001). Sensitivity analysis indicated that dietary protein's association with AD was more strongly driven by atopic sensitisation than by clinical eczema symptoms alone. Similarly, high‐GI food intake was significant associated with AD only when both clinical symptoms and atopy status were present.

Examining macronutrient intake in combination is essential to understanding the complexities of dietary influences on AD. To address this, we conducted a three‐way interaction analysis by stratifying participants into eight dietary categories based on their macronutrient intake scores. Limiting analysis to eight dietary categories preserve sufficient group sizes, ensuring statistical power. The lowest AD odds was observed with infrequent intake of all macronutrients (AOR: 0.526; 95% CI: 0.445–0.623; adjusted *p*‐value < 0.001). However, maintaining low levels across all macronutrient was not necessary; occasional intake of high‐fat and high‐protein foods alone sufficiently lowered AD odds, regardless of GI intake (AOR: 0.688; 95% CI: 0.571–0.828, adjusted *p*‐value < 0.001) (Figure 1). Synergy factor analysis showed that the macronutrient effects were independent, suggesting that lowering any single macronutrient significantly reduces AD odds without requiring moderation across all three.

**FIGURE 1:**
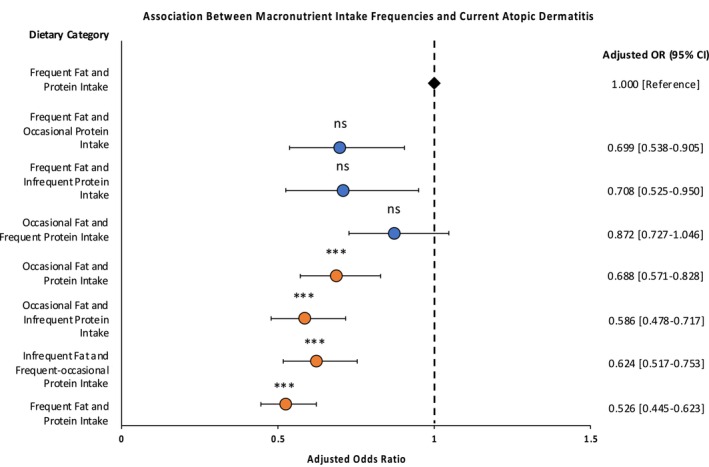
Odds ratio plot illustrating the association between three‐way intake (high‐fat, high‐protein and high‐GI foods) and current AD among 17,060 young adults from the SMCGES. Adjusted odds ratios (AOR) with 95% confidence intervals (CI) are shown. High‐GI food intake (categorised as infrequent‐frequent) was also included in the multivariable logistic regression model, though not shown as a separate visual element in the figure. The analysis was adjusted for age, sex, body mass index (Asian class), parental AD, parental education, alcohol intake and energy intake. The reference category (frequent intake for all macronutrient) is indicated by the black diamond marker, with a dashed vertical line at AOR = 1.000 for reference. Asterisks indicate statistical significance after Bonferroni correction: *p* < 0.00625 (***) with non‐significant (ns) results marked accordingly. AOR with statistical significance were marked in orange.

Our findings support a balanced approach, challenging extreme dietary paradigms that advocate severe restriction or elimination of specific macronutrients. Occasional high‐fat and high‐protein intake may be sufficient to lower the odds of current AD, providing a practical and sustainable dietary strategy for long‐term AD management. This moderation‐based approach aligns with the growing recognition in public health that a nutrient‐dense diet, focused on balance rather than exclusion, is essential in managing chronic diseases [8].

We also acknowledge the importance of differentiating between processed and fresh foods in relation to health outcomes. To enhance our findings, we seek to cross‐validate the dietary data from our simplified ISAAC FFQ with the detailed, validated 163‐item semi‐quantitative FFQ from the Singapore Multi‐Ethnic Cohort [9]. This comparison will enhance the robustness of our dietary assessment, aligning our analysis with established nutrition research frameworks and further validate our findings.

## Author Contributions

F.T.C. conceived and supervised the current research study. J.J.L. contributed to the study design, data analysis, literature review and interpretation of data. J.J.L. wrote the manuscript draft. M.H.L. provided expertise in the study methodology and critically revised the manuscript. J.J.L., K.R., Y‐H.S., assisted in the recruiting participants and collated the data. All authors have read and approved the final manuscript for submission.

## Conflicts of Interest

F.T.C. reports grants from the National University of Singapore, Singapore Ministry of Education Academic Research Fund, Singapore Immunology Network, National Medical Research Council (NMRC) (Singapore), Biomedical Research Council (BMRC) (Singapore), National Research Foundation (NRF) (Singapore), Singapore Food Agency (SFA), Singapore's Economic Development Board (EDB) and the Agency for Science Technology and Research (A*STAR) (Singapore), during the conduct of the study; and consulting fees from Sime Darby Technology Centre; First Resources Ltd.; Genting Plantation, Olam International, Musim Mas and Syngenta Crop Protection, outside the submitted work. The other authors declare no other competing interests. This research is supported by the National Research Foundation Singapore under its Open Fund‐Large Collaborative Grant (MOH‐001636) (A‐8002641‐00‐00) and administered by the Singapore Ministry of Health's National Medical Research Council.

## Data Availability

The data that support the findings of this study are available from the corresponding author (F.T.C.) upon reasonable request.
